# Rab11 and Actin Cytoskeleton Participate in *Giardia lamblia* Encystation, Guiding the Specific Vesicles to the Cyst Wall

**DOI:** 10.1371/journal.pntd.0000697

**Published:** 2010-06-01

**Authors:** Araceli Castillo-Romero, Gloria Leon-Avila, Ching C. Wang, Armando Perez Rangel, Minerva Camacho Nuez, Carlos Garcia Tovar, Jorge Tonatiuh Ayala-Sumuano, Juan Pedro Luna-Arias, Jose Manuel Hernandez

**Affiliations:** 1 Departamento de Biología Celular, Centro de Investigación y de Estudios Avanzados del IPN, México DF, México; 2 Departamento de Zoología, Escuela Nacional de Ciencias Biológicas del Instituto Politécnico Nacional, México DF, México; 3 Chemistry and Chemical Biology Graduate Program, Department of Pharmaceutical Chemistry, University of California San Francisco, San Francisco, California, United States of America; 4 Departamento de Ciencias Genómicas, Universidad Autónoma de la Ciudad de México, México DF, México; 5 Departamento de Ciencias Biológicas, FES-Cuautitlán Universidad Nacional Autónoma de México, México, México; University of Queensland, Australia

## Abstract

**Background:**

*Giardia* passes through two stages during its life cycle, the trophozoite and the cyst. Cyst formation involves the synthesis of cyst wall proteins (CWPs) and the transport of CWPs into encystation-specific vesicles (ESVs). Active vesicular trafficking is essential for encystation, but the molecular machinery driving vesicular trafficking remains unknown. The Rab proteins are involved in the targeting of vesicles to several intracellular compartments through their association with cytoskeletal motor proteins.

**Methodology and Principal Findings:**

In this study, we found a relationship between Rab11 and the actin cytoskeleton in CWP1 transport. Confocal microscopy showed Rab11 was distributed throughout the entire trophozoite, while in cysts it was translocated to the periphery of the cell, where it colocalized with ESVs and microfilaments. Encystation was also accompanied by changes in *rab11* mRNA expression. To evaluate the role of microfilaments in encystation, the cells were treated with latrunculin A. Scanning electron microscopy showed this treatment resulted in morphological damages to encysted parasites. The intensity of fluorescence-labeled Rab11 and CWP1 in ESVs and cyst walls was reduced, and *rab11* and *cwp1* mRNA levels were down-regulated. Furthermore, knocking down Rab11 with a hammerhead ribozyme resulted in an up to 80% down-regulation of *rab11* mRNA. Although this knockdown did not appear lethal for trophozoites and did not affect *cwp1* expression during the encystation, confocal images showed CWP1 was redistributed throughout the cytosol.

**Conclusions and Significance:**

Our results indicate that Rab11 participates in the early and late encystation stages by regulating CWP1 localization and the actin-mediated transport of ESVs towards the periphery. In addition, alterations in the dynamics of actin affected *rab11* and *cwp1* expression. Our results provide new information about the molecules involved in *Giardia* encystation and suggest that Rab11 and actin may be useful as novel pharmacological targets.

## Author Summary

The encystation process is crucial for survival and transmission of *Giardia lamblia* to new hosts. During this process, vesicular trafficking and the cytoskeleton play important roles. In eukaryotic cells, intracellular transport is regulated by proteins, including Rab-GTPases and SNAREs, which regulate vesicle formation along with recognition of and binding to the target membrane. Cytoskeletal structures are also involved in these processes. In this study, we demonstrate the participation of Rab11 in the transport of encystation-specific vesicles (ESVs). Additionally, we demonstrate that disruption of actin microfilaments affects ESVs transport. The modification of actin dynamics was also correlated with a reduction in *rab11* and *cwp1* expression. Furthermore, down-regulation of *rab11* mRNA by a specific hammerhead ribozyme caused nonspecific localization of CWP1. We thus provide new information about the molecular machinery that regulates *Giardia lamblia* encystation. Given our findings, Rab11 and actin may be useful targets to block *Giardia* encystation.

## Introduction

*Giardia lamblia* is the causative agent of Giardiasis, a diarrheal disease [1], [2]. *Giardia* exists in two forms during its life cycle, the trophozoite and the cyst. The trophozoite, which is responsible for the symptomatology of Giardiasis, resides in the small intestine, causing the loss of epithelial cell microvilli through mechanical damage [3], [4]. The cyst, which is the infective form, is responsible for the transmission to new hosts and can remain viable for two or three months in hostile environments [5]. The encystation of *Giardia* occurs after stimuli such as changes in pH or high concentrations of bile and involves shape changes, synthesis and transport of specific proteins including cyst wall proteins (CWPs) and biogenesis of ESVs [6]–[11].

Eukaryotic cells have a very dynamic system of internal membranes that is responsible for secretion, transport, release and recycling of proteins and other molecules. These processes take place in vesicles and involve cover proteins such as COPI, COPII and clathrin, membrane-docking proteins such as SNAREs and Rabs and cytoskeletal motor proteins [12]–[14]. The Rab proteins are recruited and activated in the donor membrane; they regulate the formation, transport, joining and fusion of the vesicles [15], [16]. Langford and coworkers used heterologous antibodies in *Giardia* to demonstrate that Rab1 and GDI orthologues were localized to endoplasmic reticulum and the surface of ESVs [17]. New orthologues of proteins involved in vesicular processes were reported by Marti and coworkers, who demonstrated a moderate increase in the expression of *rab11* during the encystation process and localization of Rab11 to the periphery of ESVs [18]. Rab11 protein has been associated with endocytosis, recycling processes and secretion; more recently, Rab11 has been found to translocate to the periphery in cystic forms of *E. histolytica*, suggesting it also plays a role in encystation [19].

Rab proteins have also been shown to facilitate the vectorial transport of vesicles through their strong connection with cytoskeletal motor proteins [12], [20], [21]. Furthermore, the microfilaments are dynamic structures that have been associated with vesicular traffic, endocytosis and exocytosis in mammalian and plant cells [22]–[24]. Recently, actin has been shown to play a role in ceramide endocytosis and the encystation process in *Giardia lamblia* [25], [26]. In this study, we describe the roles of Rab11 and actin during *Giardia* encystation. Our results demonstrate the participation of Rab11 and actin in ESVs transport and an indirect participation of structured actin in *rab11* and *cwp1* mRNA expression. Confocal microscopy showed that Rab11 colocalizes with actin and CWP1 in ESVs beginning in the early encystation stage as well as with CWP1 in the cyst wall. To better understand the role of Rab11 in encystation, we used a hammerhead ribozyme flanked with the antisense sequence of *rab11* mRNA to knock down Rab11 expression. This knock down of Rab11 expression resulted in displacement of CWP1 from vesicles to the cytoplasm. Our results thus provide new information about the molecular mechanisms of *Giardia* encystation.

## Materials and Methods

### Ethics statement

All the procedures for animal care and use were carried out following federal and local regulations for animal care and use (CINVESTAV-IACUC, approved by the Mexican Official Norm: NOM-062-ZOO-1999).

### Parasite culture and *in vitro* encystation

Trophozoites of *Giardia lamblia* WB were grown axenically at 37°C in glass culture tubes containing TYI-S-33 medium, pH 7.1, supplemented with 10% bovine serum and 0.5 mg ml^−1^ bovine bile [27]. Cultures were maintained by subculturing cells (0.5×10^5^ cell ml^−1^) twice a week. For *in vitro* encystation experiments, 6×10^5^ trophozoites obtained from exponentially grown cultures were incubated at 37°C for 24 h in encystation medium TYI-S-33, pH 7.8 supplemented with 10% bovine serum and 10 mg/ml bovine bile. Cells were counted for subcultures and encystation experiments using a hemocytometer.

### Effect of latrunculin A on *Giardia lamblia* encystation

To determine whether the actin cytoskeleton is involved in ESVs transport, latrunculin A (LA) (Sigma, St. Louis, MO, USA) was used to destabilize actin filaments. For encystation, 6×10^5^ trophozoites were cultured for 24 h in encystation medium containing 1 µM LA and analyzed by confocal and scanning electron microscopy. 0.1% DMSO, the LA vehicle, was used as negative control. The experiments were performed in triplicate.

### Expression and purification of recombinant Rab11 and CWP1

*rab11* (GenBank accession no. AF460175) and *cwp1* (GenBank accession no. U09330 ) genes were amplified by PCR from genomic DNA using the following sense primers: *Rab11A-s* 5′-AGA AGA gaa ttc AAA TGA CTG ACG CGT ACG ACC ATC-3′ or *CWP1C-s* 5′-AGA AGA gaa ttc AAA TGA TGC TCG CTC TCC TTG C-3′ and the following antisense primers: *Rab11B-as* 5′-TCT TCT gcg gcc gcT TCA GCA ACG CTT CTT TTG CTT AGT C-3′ or *CWP1D-as* 5′-TCT TCT gcg gcc gcT TTC AAG GCG GGG TGA GGC AG-3′. The restriction sites for EcoRI (sense primers) or NotI (antisense primers) are indicated by lowercase letters. PCR products were cloned into the pPROEX-1 expression vector to obtain pPROEX-1-Rab11 and pPROEX-1-CWP1 constructs, which were verified by DNA sequencing (Automated sequencer ABI Prism 310, Applied Biosystems, Foster City, CA, USA). Each construct was used separately to transform DH5α *E. coli* competent cells [28], and the over-expression of recombinant proteins was induced with 1 mM IPTG (Invitrogen, Carlsbad, CA, USA). Recombinant proteins were purified by affinity chromatography using Ni-NTA Agarose (Qiagen, Valencia, CA, USA) following the manufacturer's instructions. Protein concentration was determined by Bradford assay [29] and sample purity was assessed by 12% SDS-PAGE.

### Production of polyclonal antibodies

Recombinant CWP1 or Rab11 proteins were used to immunize Balb/c mice (100 µg) or Wistar rats (500 µg) via a series of intraperitoneal injections on days 0, 7 and 15. The first immunization contained an emulsified 10∶1 mixture of protein and TiterMax® (Sigma, St. Louis, MO, USA) [30]; the following challenges were performed with a 1∶1 mixture of protein and aluminum hydroxide. To test the induced antibodies, sera were assayed by western blot and ELISA [31], [32].

### Western blotting

Extracts of trophozoites or cysts (30 µg) were separated on 12% (w/v) SDS-polyacrylamide gels and subsequently electrotransferred onto nitrocellulose membranes. Non-specific binding was blocked by incubating the membranes for 1 h at room temperature in 5% non-fat dried milk in PBS containing 0.05% Tween-20 (PBS-T). The nitrocellulose membranes were then washed with PBS-T and incubated for 1 h at room temperature with a 1∶300 dilution of rat polyclonal anti-Rab11 antibody or mouse polyclonal anti-CWP1 antibody. Membranes were washed again and subsequently incubated with a 1∶8000 dilution of a peroxidase-conjugated goat anti-rat IgG secondary antibody (PIERCE, Thermo Scientific, Rockford, IL, USA) or a peroxidase-conjugated goat anti-mouse IgG secondary antibody (ZYMED Laboratories Inc, San Francisco, CA, USA) at room temperature for 1 h. After further washing with PBS-T, specific proteins were detected by chemiluminescence (Amersham-ECL, GE Healthcare Biosciences, Pittsburgh, PA USA).

### Two-dimensional electrophoresis

Extracts of trophozoites (40 µg) were prepared for two-dimensional analysis following the manufacturer's protocol (Bio-Rad ReadyStrip IPG Strip). IPG Strips (non-linear 3–10, Bio-Rad, Hercules, CA, USA) were used for isoelectric focusing. Rab11 protein was identified by western blotting. Sequences of *Giardia* Rab proteins were analyzed to determine the theoretical isoelectric point (pI) of each protein using the ExPASy Proteomics Server. These sequences can be found online using the following accession numbers: Rab1a (GenBank accession no. EES98506), Rab2 (GenBank accession no. XP_001707297), Rab11 (GenBank accession no. XP_001708893), Rab32 (GenBank accession no. XP_001708910), RabA (GenBank accession no. AAL87243), RabB (GenBank accession no. XP_001703990), RabD (GenBank accession no. XP_001706877) and RabF (GenBank accession no. XP_001705767).

### Immunofluorescence

Parasites were washed twice with PBS and fixed for 1 h at 37°C with 3.7% paraformaldehyde in PBS. Fixed cells were washed twice with PBS and allowed to attach to poly-L-lysine-coated cover slips. The cells were then permeabilized for 30 min with a mixture of 0.5% Triton ×100 and 0.5% SDS, washed twice with PBS and incubated for 30 min with 1% BSA to block non-specific binding. The cells were next incubated with rat polyclonal anti-Rab11 and/or mouse polyclonal anti-CWP1 antibodies (each 1∶300) for 1 hr. After further PBS washes, the cells were incubated with FITC-conjugated anti-rat (1∶50) (ZYMED Laboratories Inc, San Francisco, CA, USA) and/or Cy5-conjugated anti-mouse (1∶100) (Jackson ImmunoResearch Laboratories, West Grove, PA, USA) secondary antibodies and TRITC-phalloidin (1∶100) (Sigma, St. Louis, MO, USA) at room temperature for 1 h. Cover slips were mounted with Vectashield mounting medium (Vector Laboratories Inc., Burlingame, CA, USA) and analyzed by confocal microscopy (CM) (Leica DMIRE2 and TCS-SPE, Leica Microsystems CMS GmbH, Mannheim, Germany). Images were processed using Leica Lite and Leica Application Suite software and converted to the appropriate format.

### Scanning electron microscopy (SEM)

Parasites were washed with PBS and fixed for 1 h with 2.5% glutaraldehyde in PBS. Cells were then allowed to adhere to poly-L-lysine-coated cover slips. Adherent cells were washed three times with PBS and post-fixed in 1% osmium tetroxide for 1 h. Next, cells were washed with PBS, dehydrated in a series of increasing concentrations of alcohol (60–100%), subjected to critical point drying and coated with a thin layer of gold. The samples were analyzed by SEM (JSM-35C, JEOL Ltd. Tokyo, Japan).

### Relative-quantitative RT-PCR

Total RNA was obtained from trophozoites and cysts using TRIzol reagent (Invitrogen, Carlsbad, CA, USA). cDNA was synthesized by a reverse transcriptase reaction (Invitrogen, Carlsbad, CA, USA) using 2 µg of RNA and Oligo dt_20_ primer (Invitrogen, Carlsbad, CA, USA). Primers for RT-PCR were designed using the following GenBank *Giardia* sequences: *rab11* (GenBank accession no. AF460175), *actin* (GenBank accession no. AAA99305.1) and *cwp1* (GenBank accession no. U09330). *rab11*, *actin* and *cwp1* primer sequences were as follows: forward, *Rab11L-s* 5′-CGC GGA TCC GCG ATG ACT GAC GCG TAC GAC CAT C-3′, *ActinE-s* 5′-AGA AGA GAA TTC AAA TGA CAG ACG ACA ACC CTG CCA TAG-3′ and *CWP1C-s* 5′-AGA AGA GAA TTC AAA TGA TGC TCG CTC TCC TTG C-3′; reverse, *Rab11B-as* 5′-TCT TCT GCG GCC GCT TCA GCA ACG CTT CTT TTG CTT AGT C-3′, *ActinF-as* 5′-TCT TCT GCG GCC GCT TCA CAT ACA CTT ACG GTT TGC AAT G-3′ and *CWP1G-as* 5′-TCT TCT CCA TGG TAG GCG GGG TGA GGC AGT ACT CTC CGC AGT CCG-3′. Relative-quantitative RT-PCR was performed in a 7500 Real Time PCR System (Applied Biosystems, Foster City, CA, USA) using Maxima SYBR Green qPCR Master Mix (2×) (Fermentas Life Sciences, Burlington Ontario, Canada) to evaluate the amplification reaction. The expression levels of the above mentioned genes were normalized to the expression level of glyceraldehyde 3-phosphate dehydrogenase gene (*gap1*) (GenBank accession no. M88062) using the following primers: *gap1-s* 5′-GCA AGC GTG TCA TCA TCT CCG CTC CG-3′ and *gap1-as* 5′-AAG GAC CTT CCC GAC AGC CTT TGC G-3′. A melting curve was performed to confirm the absence of primer dimerization. The relative-quantitative RT-PCR conditions were as follows: hot start at 95°C for 10 min and 40 cycles of 95°C for 30 s, 65°C for 30 s and 72°C for 1 min. The comparative ΔΔCt method was used to calculate changes in expression [33]. Significant differences (defined as p<0.05 and indicated by asterisks in figures) were calculated by Student's *t* tests and ANOVA tests using the program GraphPad Prism 5.02. Error bars indicate standard deviations for experiments with more than one trial.

### Hammerhead ribozyme

**Figure 1:**
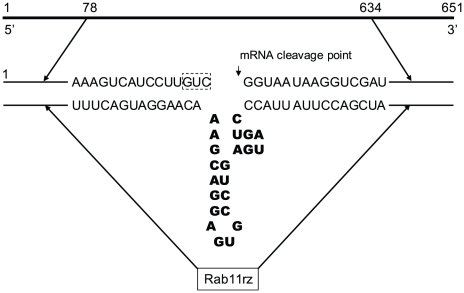
Schematic representation of the Rab11 hammerhead ribozyme. Bold letters show the ribozyme structure, the dotted box indicates the nucleotides selected as a cutting site.

Rab11 mRNA (GenBank database accession number XM_001708841) consists of 651 nucleotides. A trinucleotide GUC located in the central region (residues 373–375) was selected as the target point for ribozyme cleavage. The ribozyme designed for Rab11 (Rab11rz) included 22 nucleotides termed the ribozyme motifs [34], [35]. Rab11rz theoretically cleaves Rab11 mRNA between nucleotides 375 and 376 (Figure 1).

To obtain Rab11rz, we performed two PCR reactions using genomic *Giardia* DNA as a template. The first PCR was performed using the following primers: *Rab11H-s* 5′-AGA ctc gag GAG GTT CAC CAG CAA CAA GTT C-3′ and *Rab11I-as* 5′-AAG TGC CTG AGA TCG ACC TTA TTA CCC TGA TGA GTC CGT GAG GAC GAA ACA AGG ATG ACT TTG CAG GCC GTG GAG-3′. For the second PCR we used the following primers: *Rab11J-s* 5′-GTA ATA AGG TCG ATC TCA GGC-3′ and *Rab11K-as* 5′-TCT ctc gag GCT TAG TCT TTG CTG CAT TAT TAA-3′. The underlined sequences correspond to the core structure of the hammerhead ribozyme, and the lowercase letters indicate XhoI restriction sites.

Two products sharing an overlap region of 21 bp were amplified by these PCR reactions, a 345 nt fragment consisting of Rab11 residues 78–401 plus the ribozyme motifs and a 257 nt fragment consisting of Rab11 residues 377–634. The final product, Rab11rz, was obtained by using the above fragments as templates in a subsequent PCR with the primers *Rab11H-s* and *Rab11K-as*.

To obtain the pC631pac-Rab11rz construct, Rab11rz was digested and cloned into the pC631pac plasmid, which was linearized with XhoI. Clones containing the antisense sequence were confirmed by sequencing. The *rab11* gene was also amplified by PCR using the primers *Rab11H-s* and *Rab11K-as*. This PCR fragment was cloned into the pGEM-T vector to generate a pGEM-T-Rab11 construct that expressed the *rab11* transcript; this construct was used to test ribozyme activity.

### *In vitro* ribozyme cleavage of Rab11 mRNA

The ribozyme (pC631pac-Rab11rz) DNA was linearized with NruI and used as a template for *in vitro* transcription using the MEGAscript T7 kit (Ambion, Applied Biosystems, Foster City, CA, USA). Likewise, pGEMT-Rab11 was linearized with SalI, and a fragment of 651 nt was obtained by *in vitro* synthesis with the MEGAscript T7 kit. This 651 nt fragment was used as a substrate for ribozyme cleavage assays. The fragment was mixed with the chimeric ribozyme construct at a 1∶3 ratio in a reaction mixture containing 50 mM Tris-HCl, pH 8.0. The reaction was incubated at 95°C for 3 min and then 37°C for 5 min. Next, the mixture was centrifuged briefly at room temperature and incubated at 37°C for another 5 min. Finally, to start the cleavage reaction, MgCl_2_ was added at a final concentration of 10 mM. The reaction was continued for 3 h at 37°C, and the product was analyzed by 7% PAGE containing 7 M urea and stained with ethidium bromide.

### Transfection of *Giardia lamblia* trophozoites

The chimeric ribozyme was used to transfect GLV-infected *Giardia lamblia* WB trophozoites. Trophozoites were incubated for 30 min in ice water, centrifuged at 800×g for 10 min and washed three times with cold PBS and once with complete cytomix buffer (CyB) [10 mM K_2_HPO_4_-KH_2_PO_4_ (pH 7.6), 120 mM KCl, 0.15 mM CaCl_2_, 25 mM N-2-hydroxyethylpiperazine-N9-2-ethanesulfonic acid (HEPES), 2 mM ethylene glycol-bis(b-aminoethylether)-,N,N9,N9-tetraacetic acid (EGTA), 5 mM MgCl_2_, 2 mM ATP and 4 mM glutathione] [36]. Trophozoites (1.3×10^7^–2.3×10^7^ cells ml^−1^) were then suspended in 450 µL of CyB, placed into one 0.4 cm electroporation cuvette and kept on ice. The *in vitro*-synthesized RNA (50–100 mg) was mixed with 100 to 150 µg of yeast tRNA (GIBCO/BRL, Invitrogen, Carlsbad, CA, USA). The RNA solution was added to trophozoites immediately prior to electroporation. Standard conditions for electroporation were as follows: 500 V/cm and 500 µF with a Gene Pulser (augmented with a capacitance extender; Bio-Rad, Hercules, CA, USA). After electroporation, the cuvettes were kept on ice for an additional 10 min. Next, cells were transferred to fresh TYI-S-33 medium and incubated at 37°C for 24 h, after which 10 µg ml^−1^ puromycin was added to the culture medium. Growth was monitored and after four days the medium was replaced with fresh medium containing 20 µg ml^−1^ puromycin. Stably transfected cells were obtained through serial passaging. To induce a high level of expression of the ribozyme, puromycin was applied at concentrations of 50, 100 and 200 µg ml^−1^.

### Relative-quantitative RT-PCR assay for Rab11 expression

*rab11* mRNA expression was analyzed in *Giardia* transfected with the ribozyme by relative-quantitative PCR using the primers *Rab11L-s* and *Rab11B-as* following the procedures described above.

## Results

### Rab11 is upregulated and redistributed during *Giardia* encystation

**Figure 2:**
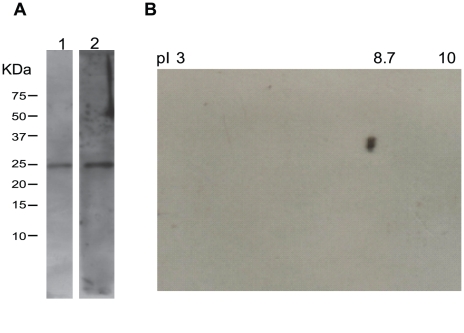
Immunodetection of Rab11 in *Giardia lamblia* lysates. (**A**). Immunoblot analysis of extracts from *Giardia* trophozoites and cysts. 30 µg of each extracts were separated on 12% SDS-PAGE transferred to a nitrocellulose membranes and probed with anti-Rab11 policlonal antibody. The antibody detects a single band with a molecular weight near 24 kDa. Lanes 1 and 2 correspond to trophozoites and cysts, respectively. (**B**). Two-dimensional electrophoresis (only one spot, pI of 8.7, was identified).

**Figure 3:**
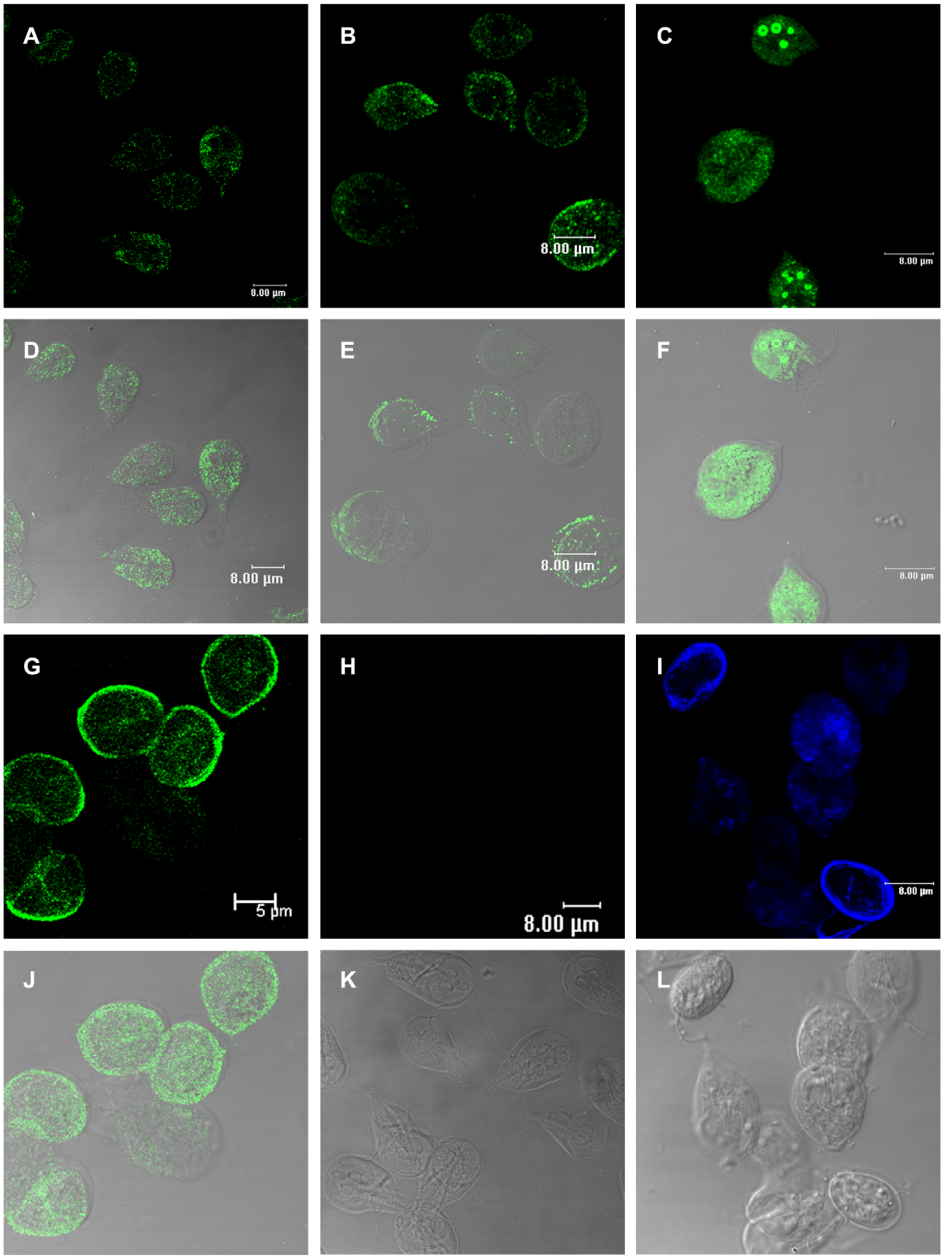
Immunolocalization of Rab11 and CWP1 during encystation of *Giardia lamblia*. Samples of trophozoites (**A**) and parasites of the different cell populations induced to undergo encystation (**B, C and G**) were processed for confocal microscopy following the protocol described in the Methods section. Images in **A–C** and **G** show cells stained with the anti-Rab11 antibody and a FITC-conjugated goat-anti rat secondary antibody. Images in **H** and **I** show trophozoites and cells induced to undergo encystation stained with the anti-CWP1 antibody and a Cy5-conjugated goat-anti mouse secondary antibody. **D–F** and **J** show superimposed images of cells stained with anti-Rab11 and corresponding DIC images. DIC images in **K** and **L** show trophozoites and cysts.

**Figure 4:**
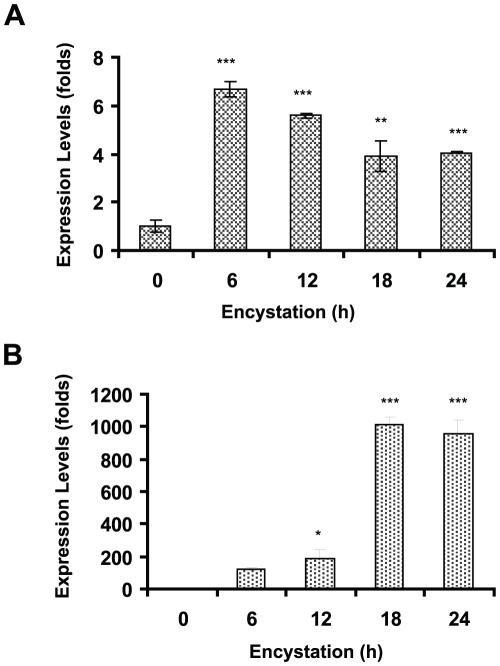
Relative-quantitative RT-PCR assay for *rab11* and *cwp1* during *Giardia lamblia* encystation. Total RNA was obtained from parasites at different times during encystation (0, 6, 12, 18 and 24 h), and *rab11*, *p* = 0.0001 (**A**) or *cwp1*, *p*<0.0001 (**B**) expression was analyzed by relative-quantitative RT-PCR analysis.

In order to detect Rab11 in both stages of *Giardia*, we produced an antibody against this protein (GenBank accession no. XP_001708893; theoretical pI/MW: 8.79/23570.71) and tested it in western blots against total lysates from *Giardia* trophozoites and cysts (Figure 2). The sera recognized a single band of the predicted size with high specificity and showed no cross-reactivity with other proteins (Figure 2A). Only one spot was identified by two-dimensional electrophoresis (Figure 2B), consistent with the Ab being specific for Rab11 and does not cross-react with any other Rabs. The distribution of Rab11 during *Giardia* encystation was analyzed by confocal microscopy using this antibody. Rab11 was visible as small light spots scattered throughout the whole body of trophozoites (Figure 3A and D). During encystation, the intensity of the staining and the protein localization changed. In precysts, Rab11 was distributed throughout the body of the parasites and in large vesicles or stacks (Figure 3B, C, E and F), while in mature cysts; Rab11 was mainly localized to the cell periphery (Figure 3G and J). To follow the encystation process, we used an anti-CWP1 antibody as a control. CWP1 was recruited into ESVs in precysts and localized to the periphery of mature cysts (Figure 3I). CWP1 was not detected in trophozoites (Figure 3H). Furthermore, during the encystation process, we measured the expression levels of *rab11* mRNA by relative-quantitative RT-PCR at 0, 6, 12, 18 and 24 h. The *rab11* mRNA reached a maximum expression level of nearly 6-fold higher than that observed in trophozoites at 6 h. Afterwards, the expression level decreased to three times that observed in trophozoites and remained almost constant from 18 to 24 h (Figure 4A). On the other hand, the *cwp1* mRNA expression began to increase during early encystation, reaching a level 120-fold greater than that in trophozoites by 6 h, and peaked between 18 h to 24 h (Figure 4B).

### Rab11 colocalizes with CWP1 and is translocated to the cell periphery

Rab11 has been associated with recycling endosomes, transport of internalized receptors and transport from Golgi to the plasma membrane.

**Figure 5:**
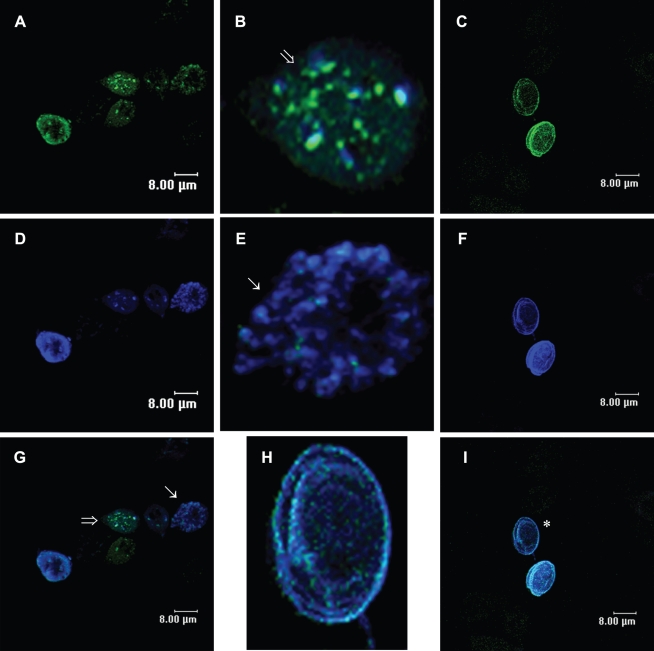
Rab11 colocalizes with CWP1 and translocates to the cyst's periphery during *Giardia lamblia* encystation. Parasites of the different cell populations induced to undergo encystation were stained with the anti-Rab11 antibody and a FITC-conjugated goat-anti rat secondary antibody (**A** and **C**) or with the anti-CWP1 antibody and a Cy5-conjugated goat-anti mouse secondary antibody (**D** and **F**). **G** and **I** are the merged images showing both Rab11 and CWP1 staining. **A** and **D** show precyst. **C** and **F** show cysts. **B**, **E** and **H** are magnified images of precysts and cysts. Arrows and the asterisk indicate the magnified cells.

Although some authors suggest that Rab11 and ESVs interact in *Giardia* [18], there are no published reports regarding the location of Rab11 in precysts and cysts. To address this issue, we performed double staining using rat anti-Rab11 (Figure 5A and C) and mouse anti-CWP1 antibodies (Figure 5D and F). The confocal images demonstrated colocalization of Rab11 with CWP1 in almost every ESVs (Figure 5B, E and G) and translocation of Rab11 and CWP1 to the cyst periphery (Figure 5H and I).

### The actin cytoskeleton colocalizes with Rab11

As mentioned above, vesicular transport is regulated by a large group of proteins and several protein–protein interactions. Previous studies demonstrated the participation of the actin cytoskeleton in intracellular transport through motor proteins [37]–[39].

**Figure 6:**
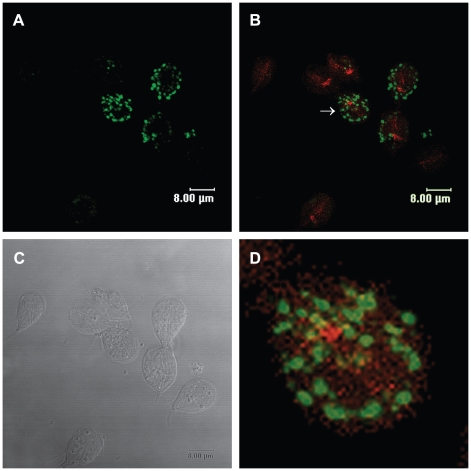
Rab11 co localizes with the actin cytoskeleton during *Giardia* encystation. (**A**)Precysts stained with the anti-Rab11 antibody and a FITC-conjugated goat-anti rat secondary antibody. (**B**) Merged image of cells stained for actin with TRITC-phalloidin and Rab11 with the anti-Rab11 antibody and a FITC-conjugated goat-anti rat secondary antibody. (**C**) DIC image. (**D**) Magnified precyst showing colocalization points between Rab11 and actin. Arrow in B indicates the magnified precyst.

Recently, we reported actin distribution throughout the whole body of trophozoites and cysts [26]. To determine whether Rab11 interacts with the actin cytoskeleton during encystation, we performed double staining. The immunofluorescence confocal images confirmed distribution of both proteins throughout the body of precysts, while Rab11 also formed aggregates (Figure 6A). The superposition of Rab11 and actin images showed sites of colocalization of these proteins (Figure 6B and D).

### The actin cytoskeleton affects parasite morphology and ESVs transport and indirectly affects *rab11* and *cwp1* gene expression

**Figure 7:**
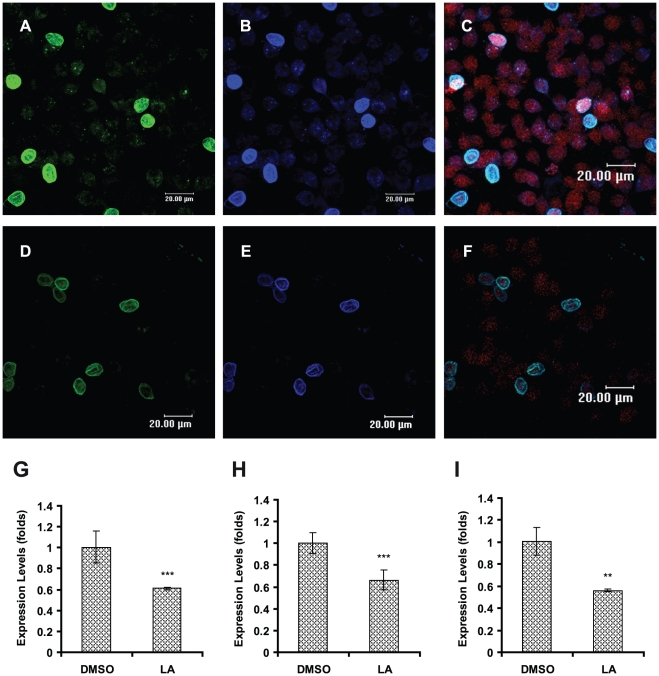
LA affects the staining of Rab11 and CWP1 and causes a down-regulation of their mRNAs during encystation. Cells induced to undergo encystation in the presence of 1 µM LA for 24 h (**D–F**) and cells induced to undergo encystation in the presence of 0.1% DMSO (**A–C**) were processed for confocal microscopy and stained with anti-Rab11 (green), anti-CWP1 (blue) and TRITC-phalloidin (red). Total RNA from these cultures was isolated and the expression of *rab11* (*p* = 0.0033) (**G**), *cwp1* (*p*<0.0001) (**H**) and *actin* (*p* = 0.0011) (**I**) was analyzed by relative-quantitative RT-PCR.

In a previous report, we demonstrated the participation of actin microfilaments in the encystation of *Giardia lamblia* [26]. To determine whether the actin cytoskeleton participates in the transport of ESVs, we induced *in vitro* encystation in the presence of 1 µM LA over a period of 24 h. The samples were processed by confocal microscopy using TRITC-phalloidin, anti-CWP1 antibody and/or anti-Rab11 antibody. LA-treated parasites showed reduced Rab11 and CWP1 staining (Figure 7D and E) concurrent with reduced staining of actin (Figure 7F) in comparison to parasites treated with DMSO as a vehicle control (Figure 7A–C). In addition, relative-quantitative mRNA analysis demonstrated that LA treatment caused a reduction in the mRNA expression level of *rab11* and *cwp1* (39% and 36%, respectively), and these decreases in expression were coincident with a reduction in the expression of *actin* mRNA (45%) (Figure 7G–I).

**Figure 8:**
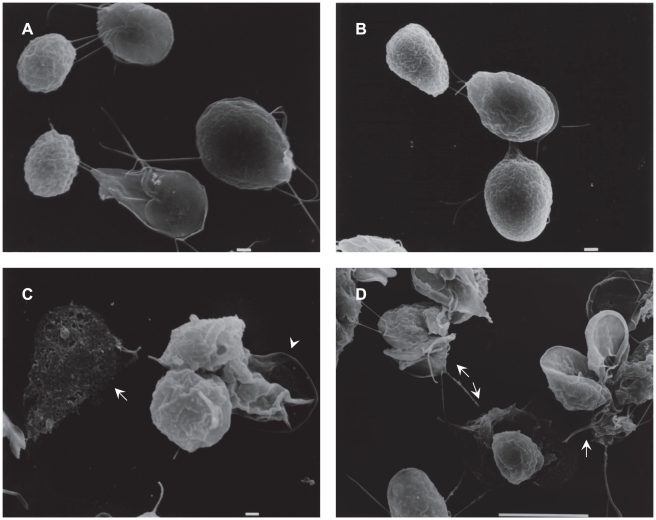
SEM micrographs of parasites treated with LA. Samples of cells induced to undergo encystation in the presence of 1 µM LA for 24 h were processed for SEM as described in the Methods section. (**C** and **D**) 1 µM LA. (**A** and **B**) 0.1% DMSO, control samples. Bars indicate 1 and 10 µm. Arrows indicate damaged cells, arrowhead indicates cysts with extracellular peritrophic space altered.

SEM analysis of LA-treated cells confirmed that morphological alterations in precysts and cysts characterized by amorphous encysting parasites with disrupted or absent flagella were caused by the drug. Several cysts exhibited alterations in size and in the extracellular peritrophic space (Figure 8C and D). Meanwhile, DMSO-treated cells showed a normal morphology, with cysts exhibiting an oval form and rough walls (Figure 8A and B).

### The hammerhead ribozyme cleaves *rab11* mRNA in an *in vitro* assay

**Figure 9:**
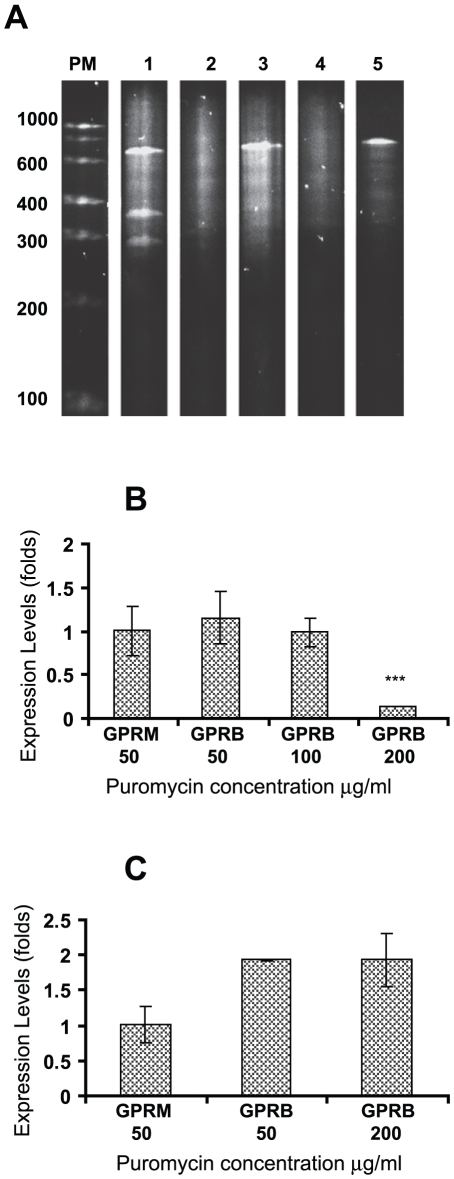
Rab11 ribozyme activity *in vitro* and *in vivo*. (**A**) “*In vitro*” cleavage of *rab11* mRNA fragment (RAF, 651 nt) by pC631pac-Rab11B and pC631pac-Rab11M. The product was analyzed on 7% PAGE containing 7 M urea and stained with ethidium bromide. Lane 1 is pC631pac-Rab11B plus RAF. Lane 2 is pC631pac-Rab11B. Lane 3 is pC631pac-Rab11M plus RAF. Lane 4 is pC631pac-Rab11M. Lane 5 is RAF. (**B**) Total RNA from cultures of *Giardia* transfected with *pC631pac-Rab11B* (GPRB) and *pC631pac-Rab11M* (GPRM) selected in 50, 100 and 200 µg ml^−1^ puromycin, was isolated and the expression of *rab11* (*p*<0.001) was analyzed by relative-quantitative RT-PCR. (**C**) The relative-quantitative RT-PCR analysis of *cwp1* gene expression in GPRB or GPRM selected in 50 and 200 µg ml^−1^ puromycin after 24 h of encystation.

Hammerhead ribozyme techniques have been successfully used in *Giardia lamblia* to determine the function of specific genes [40], [41]. In this study, we designed a ribozyme to block the translation of *rab11* mRNA (Rab11rz) (Figure 1). cDNA encoding Rab11rz was cloned into pC631pac, a viral expression vector. Two constructs, *pC631pac-Rab11B* and *pC631pac-Rab11M*, were obtained and sequenced. The alignment of these constructs with the antisense *rab11* sequence proved that only *pC631pac-Rab11B* contained the ribozyme flanked by the antisense sequence. *pC631pac-Rab11M*, which contained the ribozyme flanked by the *rab11* sense sequence, was used as a negative control (Figure S1). To test the ribozyme activity, *in vitro* transcripts of *pC631pac-Rab11B* or *pC631pac-Rab11M* were tested against a 651 nt sequence of *rab11* mRNA. *pC631pac-Rab11B*, as expected, produced two fragments of 276 and 375 nt, while *pC631pac-Rab11M* did not show any cleavage activity (Figure 9A). These results demonstrate that the antisense *rab11* sequence flanking the catalytic ribozyme center is essential for the *in vitro* cleavage of *rab11* mRNA.

### Ribozyme causes a down-regulation of *rab11* expression

To assess the “*in vivo*” activity of our ribozyme, we introduced *pC631pac-Rab11B* or *pC631pac-Rab11M* into GLV-infected *Giardia* trophozoites (designated as GPRB and GPRM, respectively) by electroporation. The presence of GLV in the transfected trophozoites ensured continuous replication and transcription of the ribozyme by the viral machinery [42]. To obtain stably transfected cells, *Giardia* transfectants were cultured in medium containing 20 µg ml^−1^ puromycin and serially passaged. The pressure of the drug was applied slowly but steadily increased (50, 100 and 200 µg ml^−1^). The expression of ribozyme in these cells was monitored by RT-PCR and was found to increase as the puromycin concentration was increased (data not shown) as previously reported by other authors [43]. The relative-quantitative RT-PCR analysis of *rab11* mRNA in these stable cultures showed up to an 80% dow- regulation of *rab11* in GPRB with cultured with 200 µg ml^−1^ puromycin (Figure 9B). GPRM did not affect *rab11* expression, and no changes were observed between GPRM and GPRB cultured with 50 or 100 µg ml^−1^ puromycin.

### Rab11 participates in the transport of CWP1

**Figure 10:**
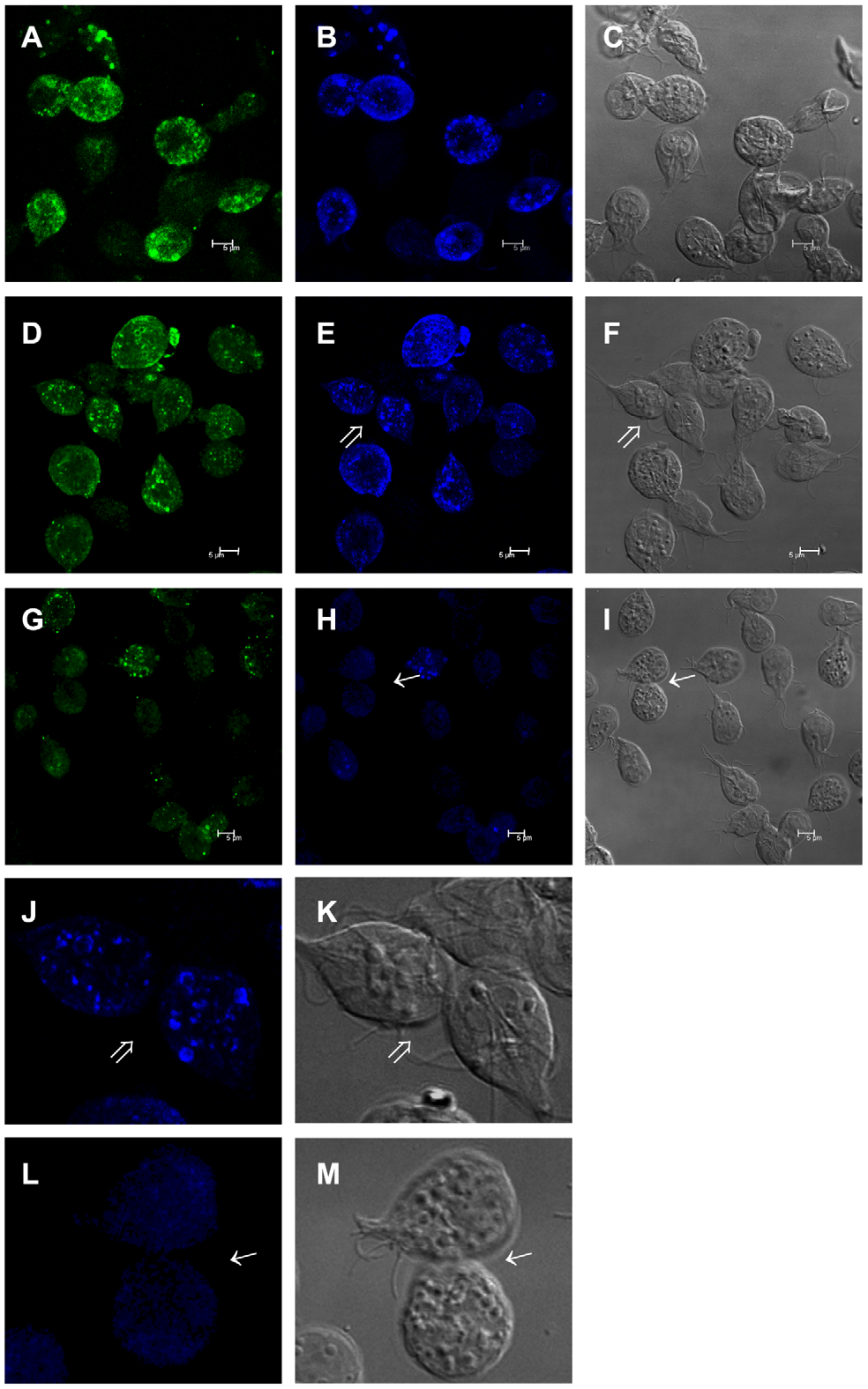
Rab11 ribozyme affected CWP1 localization during *Giardia* encystation. Samples of parasites transfected with *pC631pac-Rab11B* (GPRB) or *pC631pac-Rab11M* (GPRM) and cultured in the presence of 50 or 200 µg ml^−1^ of puromycin were induced to undergo encystation and processed for confocal microscopy. Rab11 was stained with the rat anti-Rab11 antibody and a FITC-conjugated goat-anti-rat secondary antibody (**A**, **D** and **G**). CWP1 was stained with the anti-CWP1 antibody and a Cy5-conjugated goat-anti mouse antibody (**B**, **E** and **H**). **A–C** show GPRM (50 µg ml^−1^ puromycin). **D–F** show GPRB (50 µg ml^−1^ puromycin). **G–I** show GPRB (200 µg ml^−1^ puromycin). **J** and **L** show magnified precyst. **C, F, I, K** and **M** are the DIC images. Arrows indicate magnified cells.

To determine whether the knock down of *rab11* could affect *Giardia* encystation, GPRB grown in the presence of 50 or 200 µg ml^−1^ puromycin was induced to undergo encystation for 24 h, and the expression of *cwp1* was evaluated by relative-quantitative RT-PCR. The analysis showed no significant differences in *cwp1* expression (Figure 9C); however, images obtained of these cultures by confocal microscopy showed that the knock down of *rab11* induced by 200 µg ml^−1^ of puromycin caused a reduction in CWP1 localization to ESVs. Although we observed precysts exhibiting a considerable number of ESVs, the CWP1 was mainly located in dots staining the cytoplasm (Figure 10H and L), and this change in CWP1 localization was coincident with a reduction in Rab11 staining (Figure 10G). These changes in CWP1 localization were not visible in GPRB or GPRM cultured with 50 µg ml^−1^ puromycin (Figure 10B, E, and J). Furthermore, no differences in Rab11 staining in these cells were observed (Figure 10A and D).

## Discussion

The encystation process is essential for perpetuation and transmission of *Giardia* to new hosts. In this paper, we describe the roles of Rab11 and the actin cytoskeleton during *Giardia* encystation. Using our polyclonal antibodies against Rab11 and CWP1 proteins for confocal microscopy, we demonstrated changes in the localization and distribution of Rab11 during the encystation process. Similar to the results reported in *E. histolytica* by McGugan and Temesvari, we described translocation of Rab11 to the periphery of cysts. In *E. histolytica*, Rab11 was found translocated to the periphery of the cystic forms, and the authors suggested a possible role for this protein in amoeba encystation [19]. Some authors reported minor changes in *rab11* expression during the encystation of *Giardia lamblia*; these changes were only 1-fold at 15 h and were observed by semi-quantitative RT-PCR. These authors suggested also that Rab11 could participate in encystation [18]. In this study, we confirm the participation of Rab11 throughout the entire encystation process; *rab11* expression increased during the early encystation stages, and its expression remained stable for 18–24 h.

Rab GTPases regulate intracellular traffic, and it has been hypothesized that these proteins may interact with the appropriate effectors and cytoskeletal motor proteins to perform their functions in the cell. Using drugs to disrupt the cytoskeleton in mammalian cells, some researchers demonstrated the association of Rab11 with cytoskeletal proteins to maintain appropriate distribution of proteins [44]. Furthermore, Mitra and coworkers described for the first time the participation of Rab11B in the transport and secretion of cysteine proteases in *Entamoeba* [45]; however, the function of Rab11 in *Giardia*, and specifically, the role of Rab11 and the actin cytoskeleton in ESVs transport, have remained unknown. Our results demonstrate colocalization of Rab11 with actin, suggesting that the movement of ESVs could occur through these structures.

In *Giardia*, actin was thought to function only in the attachment to glass and cell surfaces, but recently, this protein has been implicated in morphology and cytokinesis [46]–[49]. Likewise, other reports demonstrated the importance of microfilament integrity for regulated endocytic flow and for recycling of molecules to the plasma membrane [25]. Additionally, we recently described a role for actin during *Giardia* encystation based on the morphologic changes and decreased cyst yield caused by the drugs cytochalasin D, LA and jasplakinolide [26].

In the present study, we used 1 µM LA to confirm the importance of the actin cytoskeleton in *Giardia* encystation. LA treatment resulted in changes in the intensity and distribution of Rab11 and CWP1 at the ESVs and cyst wall, suggesting a novel intermolecular relationship between actin, Rab11 and CWP1. Whether this relationship is direct or indirect remains to be studied. In addition, the disruption of microfilaments resulted in down-regulation of *rab11* and *cwp1* expression. This finding is further supported by other studies in parasites and higher eukaryotes that reported that the activation of some genes is regulated by actin. Actin and its associated proteins have been shown to have a relationship with the transcription machinery [50]–[52]. Our results suggest that actin structures could regulate the expression of some genes in *Giardia*.

Ribozymes have been used successfully to determine the roles of specific genes in *Giardia* [40], [41], [53]. Previous studies have demonstrated that the cleavage of the substrate occurs after annealing by complementary recognition and the binding of the ribozyme. Binding can occur even when there are some sequence mismatches, but these mismatches must be located outside the cutting region [54]–[57]. Eight Rab proteins have been described in *Giardia*; an alignment of these proteins indicated a low identity between them (data not shown). We also performed an alignment of the antisense sequences of *rab1a* (GenBank accession no. XM_001704374), *rab2a* (GenBank accession no. XM_001707245) and *rab11* (GenBank accession no. AF460175), the *rab* genes most closely related to *rab11*, with the sequence of our ribozyme. The results showed only 24.5% identity and demonstrated that *rab1a* and *rab2a* do not contain the tri-nucleotide GUC sequence that forms the ribozyme cutting site in the same position as *rab11*. The alignments also revealed mismatches in the sequences of *rab1a* and *rab2a* near the cutting site (Figure S2). In the present study, we obtained an up to 80% knockdown of *rab11* mRNA by introducing a hammerhead ribozyme flanked by *rab11* antisense sequences. This knockdown was not lethal for trophozoites and did not affect *cwp1* expression. Nevertheless, it induced changes in the localization of CWP1 during encystation. These findings indicate that Rab11 does not regulate the expression of *cwp1*, but Rab11 and CWP1 may interact in the ESVs.

The encystation process can be divided into two phases – early and late. During the early phase, changes in structure, metabolism, gene expression and protein transport occur. The formation of ESVs occurs in the late phase. All of our results suggest that Rab11 does not contribute to ESVs formation, but it may participate in the early and late encystation stages by regulating CWP1 localization and transport of the ESVs to the periphery of the cyst through the actin cytoskeleton. It will be interesting to analyze whether Rab11 interacts also with the others CWPs.

Although neither myosin nor Rab11 effector proteins have been identified in the *Giardia* genome, it seems reasonable to consider other possible proteins that may regulate ESVs transport. Our group is currently addressing this issue. Furthermore, we cannot exclude the participation of microtubules in ESVs transport because the cytoskeleton uses both actin and microtubules during intracellular transport.

## Supporting Information

Figure S1 Alignment of *Giardia* Rab11 ribozymes against the rab11 sequence. (A) Alignment of pC631pac-Rab11B with rab11 antisense sequence. (B) Alignment of pC631pac-Rab11M with rab11 sense sequence. The red line indicates the ribozyme motifs.
(1.81 MB TIF)

Figure S2 Alignment of pC631pac-Rab11B ribozyme against *Giardia* rab11, rab1a, and rab2a antisense sequences. (A) rab11 (GenBank accession no. AF460175). (B) rab1a (GenBank accession no. XM_001704374). (C) rab2a (GenBank accession no. XM_001707245). Dotted boxes in A, B and C indicate the cutting site, and the red line in A indicates the ribozyme motifs.
(1.65 MB TIF)
