# Supplementary figures and images for: Rab11 and Actin Cytoskeleton Participate in Giardia lamblia Encystation, Guiding the Specific Vesicles to the Cyst Wall
Source: PLoS Negl Trop Dis. 2010 Jun 1;4(6):e697. doi: 10.1371/journal.pntd.0000697 (PMC2879372; doi:10.1371/journal.pntd.0000697)

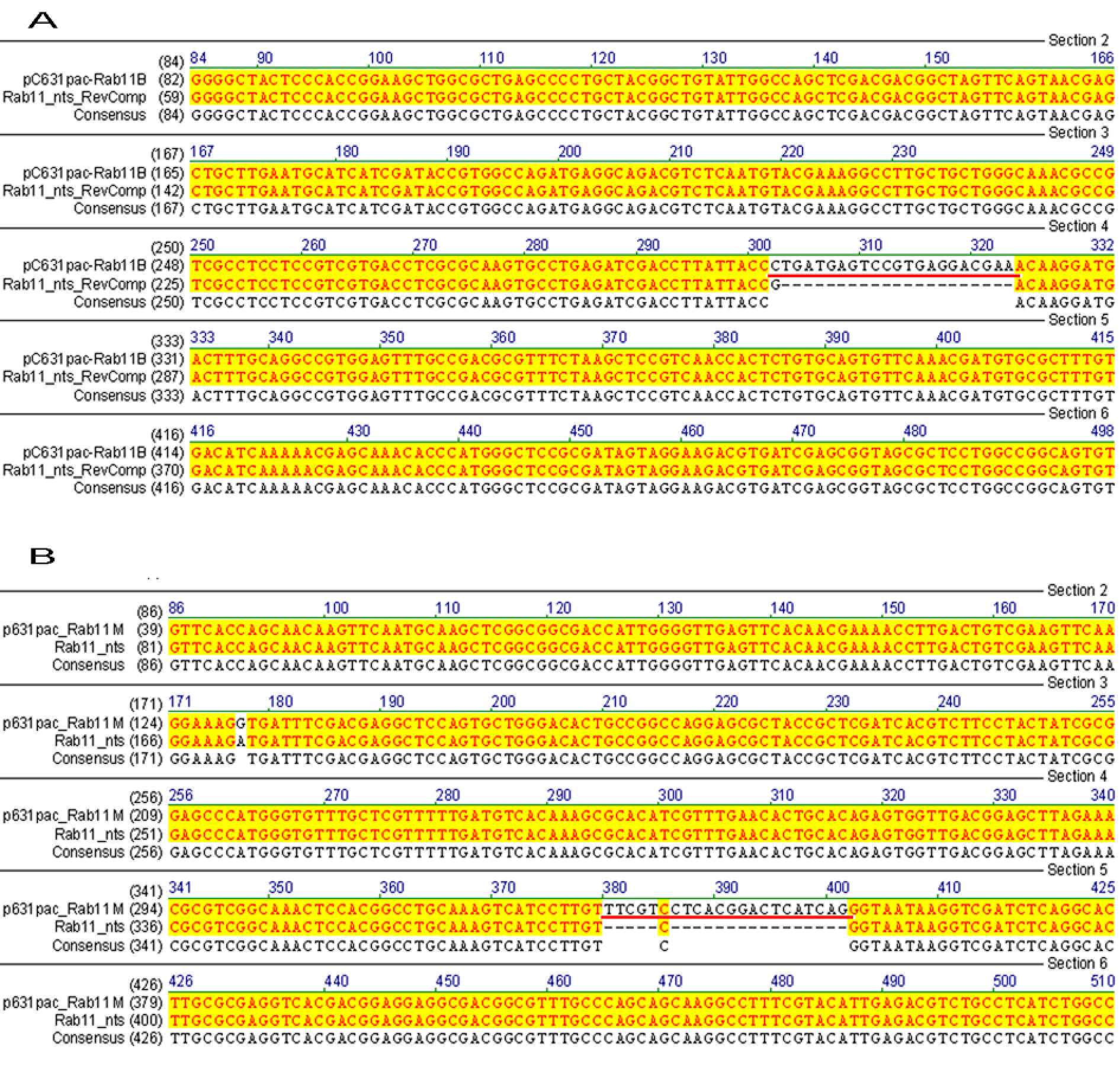

Supplement: Figure S1 — Alignment of Giardia Rab11 ribozymes against the rab11 sequence. (A) Alignment of pC631pac-Rab11B with rab11 antisense sequence. (B) Alignment of pC631pac-Rab11M with rab11 sense sequence. The red line indicates the ribozyme motifs. (1.81 MB TIF) [file pntd.0000697.s001.tif]

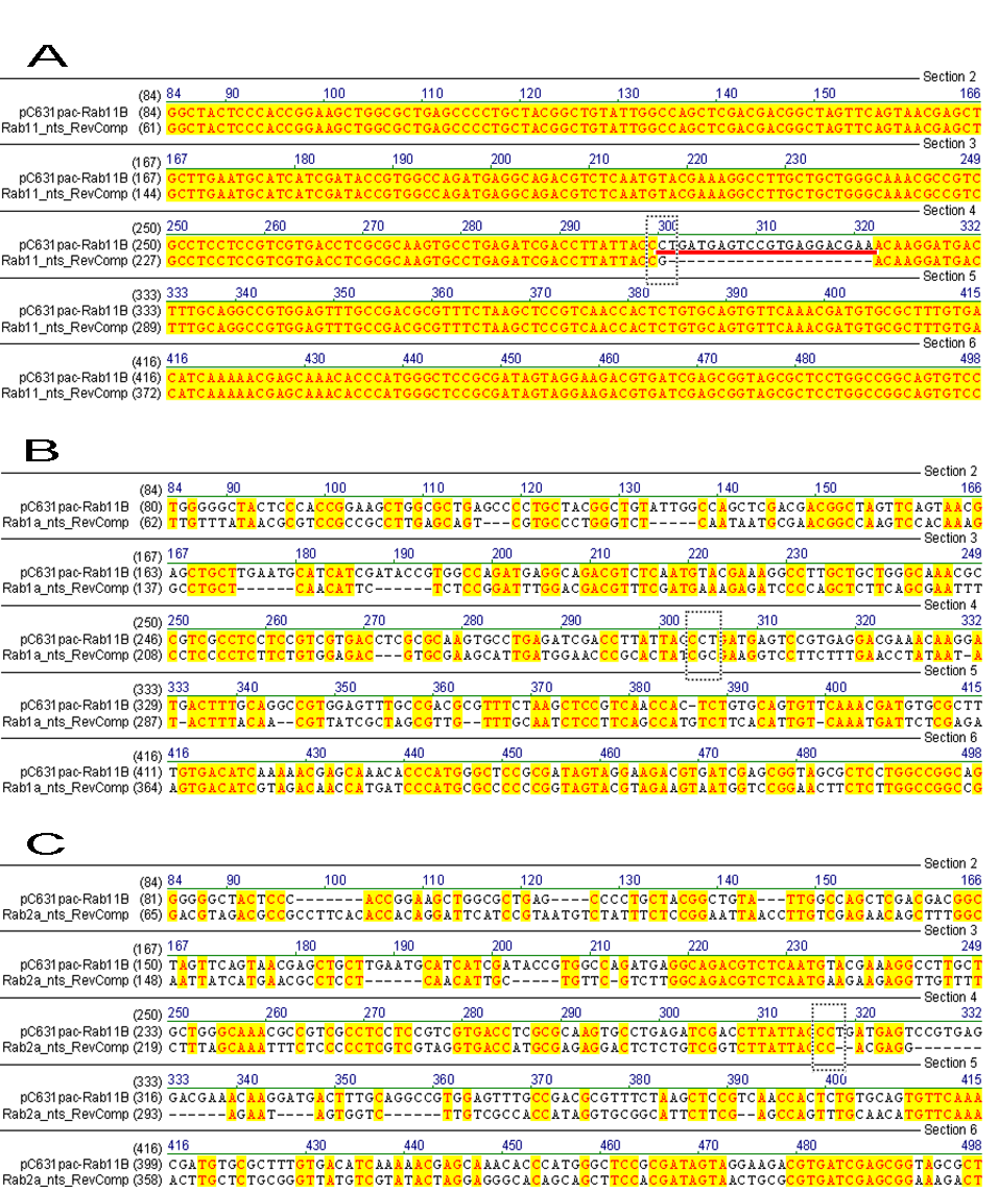

Supplement: Figure S2 — Alignment of pC631pac-Rab11B ribozyme against Giardia rab11, rab1a, and rab2a antisense sequences. (A) rab11 (GenBank accession no. AF460175). (B) rab1a (GenBank accession no. XM_001704374). (C) rab2a (GenBank accession no. XM_001707245). Dotted boxes in A, B and C indicate the cutting site, and the red line in A indicates the ribozyme motifs. (1.65 MB TIF) [file pntd.0000697.s002.tif]
